# In Vitro Models for Studying Respiratory Host–Pathogen Interactions

**DOI:** 10.1002/adbi.202000624

**Published:** 2021-05-04

**Authors:** Sarah L. Barron, Janire Saez, Róisín M. Owens

**Affiliations:** ^1^ Bioassay Impurities and Quality Biopharmaceuticals Development R&D AstraZeneca Cambridge CB21 6GP UK; ^2^ Department of Chemical Engineering and Biotechnology Philippa Fawcett Drive Cambridge CB3 0AS UK

**Keywords:** host–pathogens, immunology, in vitro models, infection, lungs, pathogens, respiratory system

## Abstract

Respiratory diseases and lower respiratory tract infections are among the leading cause of death worldwide and, especially given the recent severe acute respiratory syndrome coronavirus‐2 pandemic, are of high and prevalent socio‐economic importance. In vitro models, which accurately represent the lung microenvironment, are of increasing significance given the ethical concerns around animal work and the lack of translation to human disease, as well as the lengthy time to market and the attrition rates associated with clinical trials. This review gives an overview of the biological and immunological components involved in regulating the respiratory epithelium system in health, disease, and infection. The evolution from 2D to 3D cell biology and to more advanced technological integrated models for studying respiratory host–pathogen interactions are reviewed and provide a reference point for understanding the in vitro modeling requirements. Finally, the current limitations and future perspectives for advancing this field are presented.

## Graphical abstract

Respiratory diseases and infection are leading causes of death. This review gives a primer on biological, immunological, and pathological components occurring during respiratory infection in health and disease and provides a reference point for understanding in vitro modeling requirements. Finally, an overview of 2D, 3D, and advanced technological models are given, with prospects for the future of this field.

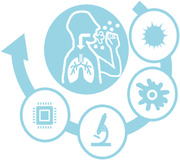

## 1 Introduction

Respiratory diseases are among the leading causes of death worldwide,^[^ ^1^ ^]^ with more than 1 billion people suffering from long standing respiratory illness.^[^ ^2^ ^]^ Among others, the most common conditions include asthma,^[^ ^3^, ^4^ ^]^ chronic obstructive pulmonary disorder (COPD),^[^ ^5^ ^]^ and idiopathic pulmonary fibrosis,^[^ ^6^ ^]^ where dysregulation, immune‐hyperresponsiveness, and remodeling of the airway epithelium is evident. In addition to chronic respiratory disease, respiratory infection also contributes a substantial burden on society, especially lower respiratory tract infections which account for 4 million deaths per annum.^[^ ^2^ ^]^ Respiratory pathogens can be highly contagious and those with underlying respiratory or immune disorders are particularly at risk of death. This area of research is highly topical given the recent Severe acute respiratory syndrome coronavirus‐2 (SARS‐CoV‐2) pandemic, which has reached over 110 749 023 confirmed cases and 2 455 131 deaths, as of February 21, 2021.^[^ ^7^ ^]^ Thus, basic pulmonary drug research and biopharmaceutical development of respiratory therapeutics, antivirals and vaccines is of paramount importance. In order to deliver the most effective treatments, however, a fundamental understanding of human lung biology is required and with it, models which accurately represent the complexity found in vivo. This is especially important when coupled to the high drug attrition rates, time to market, and ethical concerns surrounding the use of animals in research, currently seen in drug R&D pipelines.^[^ ^8^, ^9^, ^10^ ^]^ Other reviews exist with a specific focus on microfluidic^[^ ^11^ ^]^ and in silico models for drug delivery, deposition, and pharmacokinetics in preclinical lung models^[^ ^12^ ^]^ as well as ex vivo tissue engineering for lung transplantation applications.^[^ ^13^, ^14^ ^]^ In this review, however, we discuss the function and cellular composition of the pulmonary epithelium barrier—the first line of respiratory defense. We then describe the pulmonary immune system, providing a primer on its response to common respiratory pathogen, and remodeling of the respiratory epithelium in disease (asthma and COPD). We will finish by presenting the most common in vitro models for studying host‐respiratory pathogen interactions, advances in technology integrated models and future perspectives for studying these complex systems. Altogether, this review should provide the user with a basic biological understanding of the respiratory epithelial barrier and immune components required to study respiratory host–pathogen interactions in vitro. Additionally, it may be used as a reference point for understanding the requirements, relative merits, and drawbacks of using a variety of currently available in vitro lung models, ranging from 2D to complex 3D cultures. In the context of this review, 2D culture is defined as the growth of cell monolayers on a flat substrate, for example, a petri dish or polymer membrane, while 3D culture is defined as a tissue‐specific microenvironment which allows cells to retain their in vivo 3D architecture and function, for example, spheroids, organoids and use of hydrogels, scaffolds, and bioreactors.

### 1.1 The Respiratory System

**Figure 1:**
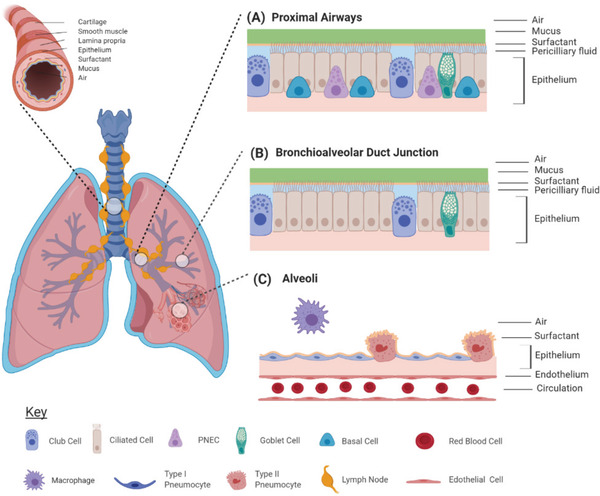
Cellular components of the lower airway pulmonary epithelium. A) The proximal (trachea, bronchi) airway epithelium consists of secretory club cells, ciliated cells, mucus producing goblet cells, basal stem cells, and pulmonary neuroendocrine cells. B) The distal portion of the lower airway consists of the bronchioles, the bronchoalveolar duct junction, and the alveoli. The bronchoalveolar duct is comprised of ciliated and club cells only. C) The alveolar epithelium consists of type 1 and type 2 pneumocytes. The blood circulation and immune cells also contribute to the defense mechanisms via interaction with the pulmonary epithelium. Image created using BioRender.com.

The human respiratory system is responsible for essential breathing processes and gas exchange. Furthermore, the pulmonary epithelium constitutes a unique interface with the outside environment, acting as a physical and immunological barrier against noxious stimuli and pathogens. Its homeostatic functions include the dynamic regulation of ion permeability, transport of essential nutrients and antimicrobial secretion.^[^ ^15^ ^]^ The respiratory system can be divided into the upper (nasal cavity, pharynx, and larynx) and lower airways (trachea, bronchi, bronchioles, alveoli, and lung parenchyma) (**Figure** **1**). The lower airway can then be further sub‐divided into three zones, according to the cellular phenotypes present: The proximal airway (trachea and bronchi) (Figure 1A), the bronchoalveolar duct junction (Figure 1B) and the alveoli (Figure 1C). The proximal airway consists of a mucus layer, a thin surfactant layer, a periciliary layer, and the epithelial layer. Mucus consists of water (97%) with small amounts of lipids, carbohydrates, and proteins.^[^ ^16^ ^]^ The most abundant proteins are mucins, secreted by goblet cells or submucosal glands, which give the mucus a gel like consistency and overall negative charge.^[^ ^16^ ^]^ These characteristics aid in capturing inhaled particles, toxins and pathogens, which are cleared from the respiratory tract via the coordinated beating of cilia (mucociliary clearance). Beneath the mucus, in contact with the cilia, is the periciliary fluid layer which contains anti‐microbials, anti‐virals, and anti‐fungals.^[^ ^17^ ^]^ Surfactant is an amphiphilic layer located between the mucus and periciliary fluid, containing predominantly phospholipids and cholesterol, with its main role to reduce surface tension and increase respiratory compliance. Surfactant is secreted in small amounts by club (formerly Clara) cells in proximal airways, although its major source of secretion is from alveolar type 2 pneumocytes.^[^ ^17^ ^]^

In addition to the aforementioned cell types, the proximal epithelium also comprises basal (stem) cells and pulmonary neuroendocrine cells (PNEC). Basal cells are responsible for epithelial regeneration upon damage, while PNECs are involved in neuroendocrine, exocrine, and immune signaling. The epithelium not only regulates selective permeability, but also homeostatic levels of hydration by active transport through the epithelial sodium channel, the cystic fibrosis transmembrane conductance regulator channel, and the calcium activated chloride channel.^[^ ^18^ ^]^ In contrast to proximal regions, the alveolar epithelium contains no ciliated cells nor does it secrete mucus, as this would reduce efficiency of gas exchange across the air‐blood barrier.^[^ ^19^ ^]^ Instead, a surfactant fluid layer together with alveolar macrophages are responsible for the protection against inhaled irritants.^[^ ^20^, ^21^ ^]^ The Alveolar epithelium consists of type 1 and type 2 pneumocytes, responsible for gas exchange and surfactant secretion, respectively. Type 2 cells also retain inducible progenitor cell properties and, if the alveolar epithelium is damaged, can differentiate into type 1 cells.^[^ ^22^ ^]^

### 1.2 The Respiratory Immune System

It is increasingly recognized that the respiratory immune system plays a fundamental role in maintaining epithelial barrier integrity and lung homeostasis, with disruption leading to the development of inflammation and disease. Additionally, the immune system contributes to barrier and protective functions through the continuous sampling of the airway lumen for non‐harmful, immunogenic, or pathogen derived antigens.^[^ ^23^ ^]^ **Table** **1** summarizes the main airway epithelial and immune cell types responsible for epithelial barrier integrity and protection, with a brief description below.

**Table 1:** The main airway epithelial and immune cell types responsible for epithelial barrier integrity and protection

Airway epithelial cell	Epithelial barrier cell function	Location in respiratory tract	Pathogen defense role
Goblet cell	Mucin production	Proximal, distal airways and submucosal glands.	Mucin directly binds/traps pathogen and cell debris; Initiates microbial phagocytosis.^[^ ^42^, ^43^ ^]^
Clara cell	Surfactant production	Proximal and distal airways.	Surfactant directly binds/traps pathogen and cell debris; activates immune cells; initiates opsonization for pathogen clearance; Initiates microbial phagocytosis.^[^ ^44^, ^45^ ^]^
Ciliated cell	Ciliary movement and clearance of mucus	Proximal and distal airways.	Involved in the Muco‐ciliary clearance mechanism and physical removal of cell debris and pathogens from respiratory tract.^[^ ^46^ ^]^
Alveolar type 2 cell	Surfactant production and inducible progenitor for type 1 alveoli cells	Alveoli.	Surfactant directly binds/traps pathogen and cell debris; activates immune cells; initiates opsonization for pathogen clearance; Initiates microbial phagocytosis.^[^ ^44^, ^45^ ^]^
Airway Immune cell	Immune component	Location in respiratory tract	Pathogen defense role
Dendritic cells (DC)	Innate immune system	Conducting airways and alveoli. Send extensions trough mucosal epithelium to sample airway. Can migrate to regional lymph nodes, once activated.	Local non‐specific inflammation; Detection of antigens; antigen presentation and priming of adaptive immune response.^[^ ^35^, ^47^ ^]^
Neutrophil	Innate immune system	Conducting airways and alveoli.	Phagocytosis; release of cytotoxic granules and neutrophil extracellular traps for pathogen entrapment; promotes recruitment of adaptive and innate immune system.^[^ ^30^ ^]^
Natural killer (NK) cell	Innate and adaptive immune system	Conducting airways and alveoli.	Directly binds infected cells and promotes lysis/apoptosis; releases cytotoxic granules; promotes adaptive immune response.^[^ ^27^ ^]^
Macrophage	Innate immune system	Alveoli (90%) and conducting airways (10%). Quiescent macrophages attach to epithelial cells, activated macrophages circulate in airways.	Quiescent macrophages suppress the overstimulation of immune system; activated macrophages secrete cytokines, stimulate dendritic cells and phagocytose cell debris and pathogens; can also present antigens in some cases.^[^ ^20^, ^29^ ^]^
T‐cell	Adaptive immune system. Naïve T‐cells can differentiate into regulatory, helper, cytotoxic or memory T‐cells	Naive T‐cells located in lymph nodes and lymph tissue. Once activated, can circulate throughout airways and alveoli.	Regulatory T‐cells suppress the overstimulation of immune system; Helper T‐cell, for example, CD4+T regulate the adaptive immune response, especially B‐cells and macrophages; cytotoxic T‐cell, for example, CD8+ bind and lyse infected cells; memory T‐cells remain and circulate after infection to ensure rapid response to reinfection.^[^ ^35^, ^37^, ^48^ ^]^
B‐cell	Adaptive immune system. Naïve B‐cells can differentiate into plasma cells or memory B‐cells	Naive B‐cells located in lymph nodes and lymph tissue. Once activated, can circulate throughout airways and alveoli.	Plasma cells secrete specific antibodies which neutralize pathogens or bind and lyse infected cells; memory B‐cells remain and circulate after infection to ensure rapid response to reinfection.^[^ ^38^, ^49^ ^]^

**Figure 2:**
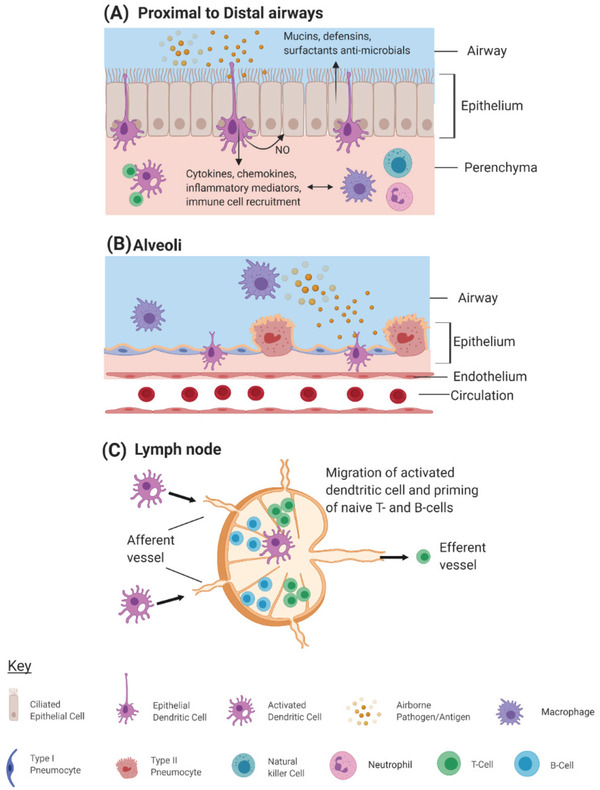
Respiratory immune cell activation in response to pathogen invasion. Airway epithelial cells (AECs) and dendritic cells (DCS) continually sample airway lumen for either airborne pathogens or allergens. Activation of specific pattern‐recognition receptors on the surface of DCs initiate an inflammatory cascade in the early stages of pathogen invasion, inducing chemokine, cytokine, and immunoregulatory compound, for example, nitric oxide (NO) production. A) Adaptive immune cells are also recruited to the site of infection and contribute to the inflammatory response as well as modulating the adaptive immune response. B) In the absence of mucus producing epithelial cells in the alveoli (which would otherwise slow down gas exchange), respiratory macrophages are the main resident immune cell type, performing a protective and phagocytic role. C) Antigen presenting DCs migrate to the lymph nodes, located throughout the proximal and distal lung regions, and prime naïve adaptive immune cells. Activated B‐ and T‐cells then migrate to the site of infection or remain in peripheral circulation as memory cells. Image created using BioRender.com.

As mentioned, airway epithelial cells (AECs) provide a physical barrier against the environment, but these cells also secrete a range of effector and regulatory molecules. These may take the form of mucins and surfactant proteins, which directly bind infectious agents and cell debris,^[^ ^23^ ^]^ or reactive species, such as nitric oxide (NO), which may influence smooth muscle contraction^[^ ^24^ ^]^ and activation of the adaptive immune response (**Figure** **2**A,B).^[^ ^23^ ^]^ AECs and dendritic cells (DC) display a range of specialized receptors capable of detecting self from non‐self antigens.^[^ ^25^ ^]^ Activation of these specific pattern‐recognition receptors initiates various immunogenic and pathogen clearance mechanisms including the early inflammatory response, recruitment of innate immune cells, and activation of the adaptive immune response.

During initial stages of pathogen invasion, the early inflammatory response is driven by the production of inflammatory chemokines and cytokines alongside recruitment of neutrophils, dendritic cells, and natural killer (NK) cells to the site of infection (Figure 2A).^[^ ^26^, ^27^ ^]^ Phagocytic macrophages may also be recruited, however, the majority of respiratory macrophages reside in the alveoli (Figure 2B) rather than the conducting airways. Neutrophils, DCs, and macrophages are capable of working synergistically upon pathogen infection to promote airway inflammation, cytokine secretion, and lysis of viral‐infected cells.^[^ ^20^, ^28^, ^29^ ^]^ Additionally, these innate immune cells are involved in the modulation of the adaptive immune response via induction of T‐cells and enhancement of DC recruitment.^[^ ^30^, ^31^, ^32^ ^]^

Induction of the adaptive immune response is also propagated through the ability of DCs to undergo a phenotypic change to present antigens.^[^ ^33^, ^34^ ^]^ Indeed, in response to respiratory infection, antigen‐presenting DCs migrate to regional lymph nodes where they prime naive adaptive immune cells for differentiation and proliferation (Figure 2C).^[^ ^35^ ^]^ T‐cell populations mitigate pathogen invasion via mechanisms specific to cell phenotype, including regulatory, cytotoxic, helper, and memory T‐cell populations (Table 1). Briefly, regulatory T‐cells are responsible for homeostatic regulation of the adaptive immune system^[^ ^23^ ^]^, while cytotoxic T‐cells directly bind and lyse infected cells. Memory T‐cell population remain in blood circulation, lymphoid or lung tissues, with lung specific memory T‐helper cells contributing to viral‐mediated immunity upon reinfection.^[^ ^36^ ^]^ B‐cell populations, once primed, mitigate pathogen invasion via the production of specific antibodies, which induce lysis and apoptosis.^[^ ^37^ ^]^ Specific memory B‐cell populations also remain as long‐lived plasma cells which persist in a quiescent state in many tissues.^[^ ^38^, ^39^ ^]^ Adaptive memory immune cell populations decline over time, with the rate of decline dependent on pathogen type and environmental conditions, meaning the potential loss of immunity over time.^[^ ^40^, ^41^ ^]^

It is also important to mention a unique immune component, specific to mucosal surfaces such as the lung and gut: the mucosal immune system, also referred to as mucosal associated lymphoid tissue. In the event that pathogens evade the physical cellular barriers of the respiratory system, mucosal tissue has unique innate and adaptive immune mechanisms, similar, but separate from the peripheral lymphoid system.^[^ ^16^, ^23^ ^]^ Thus, the mucosal immune system provides an additional protective layer in respiratory infections.

## 2 Modelling the Respiratory System In Vitro

### 2.1 Cell Types

As with the study of other biological systems, murine models are the most extensively studied in respiratory homeostasis, pathology and immune regulation. Indeed rodent models offer a complete, functioning biological system. However, since the introduction of the 3Rs principles (reduce, replace, refine), originally proposed in 1959,^[^ ^50^ ^]^ together with the cosmetic testing ban of 2013,^[^ ^51^ ^]^ there have been increasing ethical concerns surrounding animal use for scientific research. Furthermore, rodent models often lack clinical translatability, with high drug attrition rates seen in many phase III clinical trials.^[^ ^8^, ^9^, ^10^ ^]^ Thus, human derived in vitro models offer an alternative for bridging the translational gap and have been increasingly researched and developed in recent years. However, given the complexity and cellular heterogeneity of the repository epithelium throughout the airways, the specific pathogen, effector location, and diseased/healthy phenotype should inform the cell types chosen to model respiratory host–pathogen interactions.

Many lung and immune‐derived cell lines are available for culture, with the most commonly used listed in **Table** **2**. Among the lung derived cell types, Calu‐3 and A549 cell lines are most widely used. Calu‐3 cells are derived from the submucosal gland of a human cancer patient, express vast numbers of goblet cell markers, differentiate into multiple cell types when cultured at the air liquid interface (ALI), and are useful for studying mucus production and mucociliary dysfunction. A549s, although commonly used to model the alveolar epithelial barrier, are derived from type 2 pneumocytes. These cells are secretory in nature and do not contribute largely to barrier formation.^[^ ^52^ ^]^ Thus, other alveolar cell models, that represent the barrier forming type 1 pneumocytes, would be better suited for permeability, diffusion or barrier disruption experiments. To date, only one cell line is available for modelling alveolar type 1 pneumocytes (hAELVi cells),^[^ ^53^ ^]^ with other attempts mainly involving the isolation and culture of type 2 cells to give type 1‐like cell phenotypes. Important to note, however, is the derivation of cell lines from cancerous tissue and their phenotypic representation of limited cell types. Thus, the use of primary cells is preferable in representing different cell type populations, signaling interplay and the patient heterogeneity found in vivo. However, primary cells are in limited supply and are more difficult to culture. The respiratory immune system must also be represented for in vitro models to fully reflect the in vivo respiratory barrier environment. Among immune‐derived cell types, the most widely used are those obtained from peripheral blood monocytes such as macrophages^[^ ^54^ ^]^ and dendritic cells.^[^ ^55^ ^]^ It is also possible to obtain tissue specific, resident immune cells such as those adhered to the epithelium or parenchyma of lung biopsies, the most commonly derived being alveolar macrophages.^[^ ^56^ ^]^ However, the process of isolating these cells is much more time consuming and complex compared with obtaining them from blood. Immune cell lines which are derived from bone barrow of cancer patients are also available and, depending on the culture and stimulant conditions, it is possible to direct their differentiation into multiple myeloid cell types. Although this pluripotency may be advantageous in obtaining and representing multiple immune cells types, the cancerous nature of their origins will likely not reflect healthy phenotypes found in vivo. The cell type(s) one chooses for modelling the lung depends on the specific application and experimental question to be addressed. Important points to consider are: Location along the respiratory track, cell phenotypes and populations to be represented, importance of epithelial barrier formation and type of cell secretion or immune‐cell signaling pathways under study.

**Table 2:** Cell types used for in vitro respiratory models

Name	Cell type	Cell origin	Use in modeling specific cell types
Respiratory system‐derived			
HNE^[^ ^57^ ^]^	Primary cell	Human primary nasal epithelial cells from patient brushings.	Nasal epithelial cells.
NHBE^[^ ^58^ ^]^	Primary cell	Primary human bronchial epithelial cells from patients.	Bronchial epithelial cells.
Calu‐3^[^ ^59^ ^]^	Cell line	Human adenocarcinoma cell line from 25‐year‐old male patient.	Bronchial epithelial cells.
16HBE140^[^ ^60^ ^]^	Cell line	Human bronchial epithelial cell line from a 1‐year old male lung/heart transplant patient.	Bronchial epithelial cells.
A549^[^ ^61^ ^]^	Cell line	Human adenocarcinoma cell line from 58‐year‐old male.	Alveolar type 2 cells.
hAELVi^[^ ^53^ ^]^	Cell line	Human alveolar epithelial cell line.	Alveolar type 1 cells.
hAEpC^[^ ^62^ ^]^	Primary cell	Isolation and culture of type 2 human carcinoma alveolar epithelial cells.	Alveolar type 2 and type 1‐like cells.
TT1^[^ ^63^ ^]^	Cell line	Transduced human type 2 carcinoma cells line (type 1‐like phenotype).	Alveolar type 1 cells.
NCl‐H441^[^ ^64^ ^]^	Cell line	Human type 2 carcinoma cell line.	Alveolar type 2 cells.
Immune system‐derived			Use in modeling disease‐associated inflammatory pathways
Macrophage^[^ ^54^ ^]^	Primary cell	Human peripheral blood monocytes.	Macrophage induced phagocytosis and inflammation.
Dendritic cell^[^ ^55^ ^]^	Primary cell	Human peripheral blood monocytes.	Dendritic cell induced inflammation.
Neutrophils^[^ ^65^ ^]^	Primary cell	Human peripheral blood.	Neutrophil induced inflammation.
Alveolar macrophage^[^ ^56^ ^]^	Primary cell	Human lung tissue or bronchoalveolar lavage fluid.	Macrophage induced phagocytosis and inflammation.
HL‐60^[^ ^66^ ^]^	Cell line	Human acute promyelocytic leukemia cell line from a 36 year old women patient.	Spontaneous and directed differentiation into neutrophilic, monocytic, eosinophilic, and macrophage phenotypes.
THP‐1^[^ ^67^ ^]^	Cell line	Human acute monocytic leukemia cell line from a 1 year old male patient.	Spontaneous and directed differentiation into neutrophilic, monocytic, eosinophilic, and macrophage phenotypes.
HMC‐1^[^ ^68^ ^]^	Cell line	Human acute systemic macrocytosis cell line.	Mast cell induced inflammation.
LADR^[^ ^69^ ^]^	Cell line	Human acute systemic macrocytosis cell line.	Mast cell induced inflammation.

### 2.2 Traditional Model Systems for Studying Respiratory Host–Pathogen Interaction

Having given an overview of the respiratory epithelial system, lung and immune cell types available, we now consider the range of traditional in vitro models available for studying the lung microenvironment, each with their own merits, drawbacks, and benefit–cost ratio. Of important consideration is the societal need for having representative, reproducible in vitro platforms for the efficient discovery of virulence mechanism and development of antiviral vaccines for any future novel pathogens. **Table** **3** gives an overview of the traditional lung models available which are also briefly discussed below.

**Table 3:** Most common in vitro respiratory models to study host–pathogen interactions

Model type	Advantages	Disadvantages	In vitro example of host pathogen interaction	Cell type(s) used
**Submerged cell line culture** 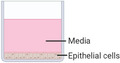	– Easy to culture. – 2–5 days culture period. – Less skill required. – Readily available/cheap.	– Representative of one cell type only. – Usually a cancerous cell line. – 2D culture. – Not representative of air interface.	Respiratory syncytial virus^[^ ^70^ ^]^	Bronchial cell line (BEAS‐2B); Primary human nasal and bronchial epithelial cells.
**ALI monoculture** 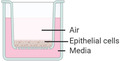	– Representative of air interfaced condition found in vivo. – Permits the study of viral entrance and metabolic pathways apically and basally.	– More expensive. – 3–4 weeks culture period with primary cells. – 2D architecture.	SARS‐CoV^[^ ^72^ ^]^	Primary human alveolar type II cells.
			SARS‐CoV^[^ ^92^ ^]^	Calu‐3 cell line.
**ALI co‐culture** 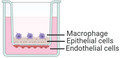	– Most biomimetic static cell culture available. – Representative of multiple cell types and systems found in vivo. – 2.5D architecture.	– High level of skill needed to culture. – 4–6 weeks culture period with primary cells.	*Aspergillus (A.) fumigatus* ^[^ ^73^ ^]^	Human primary bronchial epithelial cells, small airway cells, human blood derived macrophages, and dendritic cells.
**Polymer scaffolds** 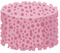	– Ability to house multiple cell types. – 3D architecture.	– High level of skill and precision needed to slice and culture. – Difficulty in monitoring cells within structure.	Influenza A^[^ ^115^ ^]^	Human primary small epithelial cells.
			Papain (mimics air bourne allergen)^[^ ^81^ ^]^	Calu‐3 epithelial cell line, MRC‐5 fibroblast cell line, blood‐derived dendritic cells.
**Organoids** 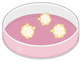	– Derived from stem cells. – Representative of the integrated tissue found in vivo. – 3D structure.	– High level of skill needed to culture. – 3–5 weeks culture period. – Cant access/monitor apical and internal cell types without disrupting.	Parainfluenza^[^ ^86^ ^]^	Human embryonic stem cells.
			Respiratory syncytial virus^[^ ^85^ ^]^	Human embryonic stem cells.
			Multiple emerging influenza virus^[^ ^91^ ^]^	Tissue resident adult stem cells.
**Precision cut lung slices (PCLS)** 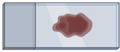	– Fully differentiated tissue. – Representative of the heterogeneous phenotypes of population. – 3D architecture. – Culture times are less than that of ALI culture.	– High level of skill and precision needed to slice and culture. – Expensive and limited supply.	Influenza^[^ ^88^ ^]^	Healthy lung slices from cancer patients undergoing lung resection.
			Rhinovirus^[^ ^89^ ^]^	Healthy and asthmatic lung slices from patient donors.
			LPS (mimics bacterial infection)^[^ ^90^ ^]^	Lung slices from patients with a variety of medical conditions from the National Disease Research Interchange.

Cartoon insets created using BioRender.com.

#### 2.2.1 2D In Vitro Models

Culturing a submerged cell line in 2D offers a relatively cheap and quick culture method (typically 3–5 days) which may be advantageous for high throughput screening and assay development. For example, a cell line may be cultured in a well plate, inoculated with an isolated virus of varying MOIs (multiplicity of infection) and plaque forming units measured from cell supernatant,^[^ ^70^ ^]^ with the whole assay taking less than a week to perform. However, submerged cell culture does not reflect the native air interface of the respiratory system which can influence the differentiation and growth processes of cell culture.^[^ ^58^, ^61^, ^71^ ^]^ Air‐interfaced cultures are most appropriate in this context, with the supply of nutrients both apically and basally during differentiation, and air lifting post‐differentiation, reflecting the environments and processes found in vivo. Additionally, ALI culture grown in a Transwell configuration provides a more in depth analysis of viral entrance. For example, it is possible to inoculate with infected serum, or using aerosol deposition atop the cell culture, mimicking entrance in vivo. Collecting cell supernatant both apically and basally, then permits the study and spatial identification of cell specific entrance with methods such as 3D immunofluorescence rendering and quantification as well as RNA extraction, viral plaque forming assays, and scanning electron microscopy.^[^ ^72^ ^]^ Culturing primary cells in Transwell configurations, and the formation of a pseudostratified epithelium, permits patient‐ and disease‐specific studies of response to infection and therapeutics in 2.5D. Multiple cell types, such as epithelial, endothelial, or immune cells can also be co‐cultured on either the basal or apical side on the Transwell filter insert, representing a more complex and complete model. Additionally, by using functional confocal microscopy and live capture video analysis, it is possible to obtain pathogen‐induced measures of immune cell recruitment, receptor entrance, and transmigration through the membrane and cell layers.^[^ ^73^ ^]^ Other gold‐standard assays involve monitoring epithelial barrier integrity during pathogen challenge using trans‐epithelial electrical resistance (TEER)^[^ ^74^ ^]^ or ionic conductance.^[^ ^75^ ^]^ However, important to note, is that primary cells are of limited supply and require a much longer culture, inoculation, and treatment period than cell lines (typically 4–6 weeks).

#### 2.2.2 Toward 3D In Vitro Models

In contrast to 2D models, 3D models more accurately represent the physiological architecture found in vivo. For example, it is possible to provide structural, mechanical, and spatiotemporal cues to the biological system, factors known to guide developmental and differentiation processes.^[^ ^76^ ^]^ Additionally, it is possible to recreate and study cell–cell and cell–extracellular matrix (ECM) interactions. An example of this, is the use of hydrogels in a range of tissue specific 3D models including lung organogenesis,^[^ ^77^ ^]^ tumorigenesis,^[^ ^78^ ^]^ and airway scaffolds.^[^ ^79^ ^]^ Hydrogels may be natural or synthetic and can be chemically, mechanically, and physically tuned to their specific application. Synthetic hydrogels are made from materials such as polyethylene glycol, polylactic acid, poly(lactic‐co‐glycolic acid), polyvinyl alcohol, and polycarprolactone. Natural hydrogels are made from a combination of polysaccharides, such as alginate, hyaluronic acid, agarose, chitosan, dextran, and cellulose, and proteins such as collagen, gelatine, fibrin, and poly‐l‐lysine (PLL). Matrigel is an example of a natural hydrogel, derived from Engelbreth‐Holm‐Swarm mouse tumor basement membranes, that is widely used in tissue culture applications. The chosen materials, however, are based on trade‐offs between biocompatibility, biodegradability, homogeneity, and mechanical durability, each having their own advantage.

#### 2.2.3 Polymer Scaffolds

Polymer scaffolds are commonly fabricated using electrospinning^[^ ^80^, ^81^ ^]^ or phase separation and freeze drying techniques.^[^ ^79^, ^82^, ^83^ ^]^ Scaffolds are seeded with lung cells and/or supporting immune and fibroblast co‐cultures, providing a biomimetic lung architecture that permits cell movement and ECM‐interaction. Furthermore, compared to 2D culture models, the use of scaffolds can increase the viability, differentiation, and expression of phenotypic markers found in vivo.^[^ ^79^, ^83^ ^]^ 3D scaffolds have been used to model respiratory infection^[^ ^115^ ^]^ and immune response to allergens in lung disease,^[^ ^81^ ^]^ with changes in epithelial barrier permeability, gene expression post‐inoculation observed. It is also possible to fix or lyse scaffolds for detailed microscopy analysis to observe any cell‐scaffold interaction.^[^ ^79^ ^]^ Although 3D polymer scaffolds pave the way for more representative models of lung tissue, they still hold some limitations such as heterogeneity of scaffold pore size and static cell culturing conditions.

#### 2.2.4 Organoids

In contrast to cellular models, organoids represent a fully differentiated 3D tissue structure. Lung organoid models are derived from human inducible pluripotent stem cells (iPSCs), embryonic stem cells, or ex vivo adult stem cells (ASCs) and may be grown at ALI or embedded within hydrogels.^[^ ^84^ ^]^ Although 3D hydrogel organoid models are largely applied to the study of developmental processes^[^ ^77^ ^]^ and signaling networks involved in the evolution of lung cancer,^[^ ^78^ ^]^ they have been increasingly used in the field of respiratory disease, virology, and drug toxicology testing. Pathogen inoculation proceeds by applying a viral solute or aerosol on top of Matrigel embedded lung organoids,^[^ ^85^ ^]^ while disease phenotypes may be induced by stimulating organoids with disease‐associated cytokine cocktails or via genetic modification of stem cells.^[^ ^85^ ^]^ In response to infection, it is possible to image, in real time, the entrance site and migration of infection both locally, within specific cell types, and globally throughout the entire lung.^[^ ^85^, ^86^, ^87^ ^]^ Furthermore, organoid models offer the advantage of being able to study cell–ECM interaction, an important consideration when evaluating immune cell and pathogen interaction within the native organ. Organoids may also be co‐cultured with human endothelial cells,^[^ ^77^ ^]^ improving biomimicry and providing an opportunity to model vasculature‐organ‐ECM interactions and virulence of pathogens. However, a limitation still remains within these complex 3D organoid models in the inability to access or monitor the apical or inner epithelium of the organoid.

#### 2.2.5 Precision Cut Lung Slices

Another 3D model, representative of the native lung tissue, is precision cut lung slices (PCLSs). In contrast to cell and organoid culturing methods that require lengthy culturing times for differentiation, PCLSs offer the advantage of retaining native tissue structure and specific macrophage populations. PCLSs have been used to study respiratory pathogen virulence^[^ ^88^, ^89^, ^90^ ^]^ as well as respiratory diseases, inflammation, and response to novel drug candidates.^[^ ^91^ ^]^ However, like human primary cells, human PCLSs are limited in supply and last in culture for an average of 7 days compared to that of 21–28 days for ALI culture. Thus, they are unsuitable for long term exposure studies.

Having given an overview and progression of traditional models for studying host respiratory processes, we now consider, in more detail, the most common types of respiratory pathogen and how host–pathogen responses may be modeled in vitro.

### 2.3 Respiratory Disease and Infection

Many chronic autoimmune and lung diseases display aberrant immune and epithelial barrier function as hallmarks of their pathology. Here, we focus on asthma and COPD which, worldwide, have the highest prevalence among respiratory diseases. Thus, understanding their underlying biomechanisms, co‐morbidities, and vulnerabilities are of high socio‐economic and therapeutic importance. Although there is evidence for asthma‐COPD overlap syndrome,^[^ ^93^, ^94^ ^]^ highlighting the complex and interconnected mechanisms underlying their pathology, here the diseases will be discussed predominately in isolation.

#### 2.3.1 Asthma

Asthma is characterized as a chronic inflammatory condition with concomitant remodeling of the proximal and distal airways.^[^ ^95^ ^]^ Although heterogeneities and subtypes exist, asthma can be broadly classified as intrinsic (non‐allergic) or extrinsic (allergic),^[^ ^96^ ^]^ with clinical presentation of exacerbation including shortness of breath, wheezing, cough and, in severe cases, airway obstruction and respiratory failure.^[^ ^95^ ^]^ Many reviews exist on the molecular, immunological, and pathological mechanism leading to remodeling of the airway epithelium in asthma^[^ ^3^, ^4^, ^95^, ^97^ ^]^; however, here we highlight models used to represent asthma in vitro.

Asthma may be modeled with the use of biopsies,^[^ ^98^ ^]^ PCLSs,^[^ ^99^ ^]^ or primary asthma cells grown at ALI.^[^ ^100^ ^]^ Co‐culture of asthmatic primary and immune cells may be used to study the crosstalk between the epithelium, immune system, and inflammatory signals in disease progression.^[^ ^100^ ^]^ Additionally, tri‐culture with epithelial, endothelial, and immune cell components may be used to represent blood vessel compartments and signaling interplay between these cell types. Perfused culture systems are also useful in mimicking the native environment under flow, and have been increasingly used to studying lung inflammation, fibrotic remodeling, and response to therapeutics.^[^ ^101^, ^102^ ^]^ Additionally, these systems may be used to study differences in healthy versus diseased airway response to environmental triggers and drugs. Indeed, application of vaporized cigarette smoke, under flow, revealed previously undiscovered disease specific molecular signatures, potentially useful for future biomarker, and drug target studies.^[^ ^103^ ^]^

Asthmatic in vitro models successfully recapitulate aspects of the airway environment found in patients, for example, displaying fewer epithelial tight junction protein complexes, increased permeability, and increased sensitivity to environmental triggers such as cigarette smoke.^[^ ^104^ ^]^ Additionally, the use of patient samples means that the heterogeneity in disease severity will be represented when subjecting these systems to infection or novel therapeutics. This is highly relevant, given that respiratory infection is a major cause of asthmatic exacerbations.^[^ ^105^ ^]^ Indeed, a body of evidence exists which argues that viral infection during childhood contributes to the initial pathogenesis of asthma.^[^ ^106^, ^107^ ^]^ Thus, modeling the interactions between the asthmatic pro‐inflammatory environment, environmental triggers, and pathogen‐specific virulence, are fundamental in understanding and treating asthmatic population with an increased vulnerability to infection.

#### 2.3.2 COPD

COPD is characterized as a progressive and chronic inflammatory disease, occurring in all parts of the lung including airways, pulmonary vasculature, and lung parenchyma.^[^ ^108^ ^]^ COPD shares some commonalties with asthma, for example, airway remodeling, chronic inflammation, and enhanced immune recruitment; however, COPD has its own defining features. In contrast to asthma, the airway remodeling that occurs in COPD is fibrotic, fixed, and irreversible^[^ ^5^, ^108^ ^]^ and, among environmental factors, smoking has the largest influence on disease progression.^[^ ^109^ ^]^ Substantial epithelial and endothelial apoptosis is present^[^ ^110^ ^]^ and, in advanced stages, COPD exacerbations can lead to hyperventilation, hypoxia, and respiratory failure.^[^ ^111^, ^112^ ^]^ Reviews exist which describe the inflammatory and molecular mechanisms behind COPD pathology in detail^[^ ^5^, ^108^, ^113^ ^]^; however, here ways to model COPD are highlighted.

Often, patient biological samples are taken in the form of blood, bronchoalveolar lavage,^[^ ^76^, ^114^, ^115^, ^116^ ^]^ biopsies,^[^ ^117^, ^118^ ^]^ and cell brushings.^[^ ^79^, ^80^, ^119^ ^]^ These are used to study levels of inflammatory markers and immune cell activity, which may also form the basis for patient stratification and treatment. It is also possible to study markers of mucociliary clearance, such as levels of ciliary metaplasia^[^ ^80^, ^120^ ^]^ and ciliary beat frequency,^[^ ^79^ ^]^ the reduction of which may increase patient vulnerability to infection and sputum production. An altered respiratory microbiota is also implicated in COPD pathophysiology,^[^ ^121^ ^]^ with acute exacerbations linked to microbial–pathogen interactions and infection.^[^ ^122^, ^123^, ^124^ ^]^ Mechanisms underlying these findings may be recapitulated using in vitro models of COPD, cultured with primary cells. For example, oxidative mechanisms,^[^ ^125^ ^]^ and an enhanced inflammatory environment^[^ ^126^ ^]^ have been shown to augment epithelial cytokine and specific recognition receptor expression in viral‐induced COPD exacerbations. Additionally, to account for the increased risk of smokers developing COPD and viral‐induced exacerbations, mechanisms behind smoke induced epigenetic changes in bronchial epithelium have also been explored, such as an increase in mesenchymal markers^[^ ^127^ ^]^ and a decrease in antiviral cytokine expression.^[^ ^128^ ^]^ These in vitro COPD models also serve the purpose of high throughput drug development for disease related complications, such as viral induced exacerbations,^[^ ^127^ ^]^ highlighting the potential for personalized therapeutics based on disease heterogeneity and severity.

So far we have considered the respiratory epithelial and immune systems in health and in diseases such as COPD and asthma. Next, we discuss specific pathogen virulence mechanisms, the altered host response to infection, and how this interplay may be modeled in vitro.

## 3 Respiratory Pathogens

The presence and accumulation of pathogens within the respiratory system can perturb homeostasis by overcoming the epithelial barrier and eliciting an immune response. Despite the overlap of symptoms and clinical manifestations of respiratory infections, individual pathogen types and species have distinct modes of entrance and virulence (**Table** **4**), with the host environment and health status influencing severity and vulnerability to infection. Respiratory infection may be modelled in vitro by inoculating the cell culture system with isolated pathogen particles or with immunostimulants which mimic pathogen‐specific inflammatory processes. Important considerations in choosing a model pathogen is whether the cell model expresses the relevant pathogen‐specific receptor, the respiratory location which the pathogen infects, and the experimental readouts you wish to use to assess virulence. Here, a brief overview of the most prevalent viral, bacterial, and fungal respiratory pathogens are given, together with their application in in vitro culture systems for studying respiratory host–pathogen interaction.

**Table 4:** The most common viral, bacterial, and fungal pathogens known to cause repository infection

Pathogen	Clinical symptoms (complications)	Respiratory tract infected part	Pathogen entrance mechanism
**Viral**			
MERS‐CoV	Fever, chills, sore throat, cough, shortness of breath, headache, vomiting, diarrhoea, myalgia (pneumonia, septic shock, severe acute respiratory distress syndrome, respiratory failure, multi‐organ failure).	Upper and lower respiratory tract.	Cell mediated membrane fusion or endocytosis via CD26 receptors.^[^ ^131^ ^]^
SARS‐CoV	Fever, chills, myalgia, shortness of breath (pneumonia, fibrosis, severe acute respiratory distress syndrome, respiratory failure).	Upper and lower respiratory tract.	Cell mediated membrane fusion or endocytosis via ACE2 receptors.^[^ ^132^ ^]^
SARS‐CoV‐2 (COVID‐19)	Fever, chills, cough, shortness of breath, sore throat, rhinorrhoea, temporary anosmia or ageusia (pneumonia, septic shock, severe acute respiratory distress syndrome, respiratory failure, multi‐organ failure).	Upper and lower respiratory tract.	Cell mediated membrane fusion or endocytosis via ACE2 receptors.^[^ ^133^ ^]^
Seasonal influenza	Fever, sore throat, cough, headache, rhinorrhoea, myalgia, headache, (laryngotracheobronchitis, bronchitis).	Upper respiratory tract.	Cell mediated membrane fusion via sialic acid containing receptors and protease cleavage.^[^ ^136^ ^]^
Respiratory syncytial virus (RSV)	Fever, sore throat, cough, headache, rhinorrhoea, shortness of breath, wheezing, (laryngotracheobronchitis, bronchitis).	Lower respiratory tract.	Cell mediated envelope fusion via nucleolin containing receptors.^[^ ^137^ ^]^
Rhinovirus	Sore throat, cough, rhinorrhoea (bronchitis).	Upper respiratory tract.	Cell mediated endocytosis via ICAM‐1, LDL or CDHR3 receptors.^[^ ^169^ ^]^
**Bacterial**			
*Streptococcus pneumoniae*	Fever, chills, cough, shortness of breath, chest pain, (pneumonia, septic shock, bacteraemia, meningitis).	Forms part of upper respiratory tract flora but can migrate and cause infection in lower respiratory tract and/or spread systemically.	Extracellular colonization; polysaccharide capsule promotes adherence and protection.^[^ ^144^ ^]^
*Haemophilus influenzae*	Fever, chills, cough, shortness of breath, chest pain, (pneumonia, bronchitis, septic shock, bacteraemia, meningitis).	Forms part of upper respiratory tract flora but can migrate and cause infection in lower respiratory tract and/or spread systemically.	Internalization by epithelial cells via micropinocytosis and rearrangement of epithelial cytoskeleton^[^ ^141^ ^]^; internalization by macrophage and neutralizes lysosomes to prevent detection or lysis.^[^ ^147^ ^]^
*Mycobacterium tuberculosis*	Fever, chills, chest pain, cough, weight loss (meningitis, respiratory failure, multi‐organ failure).	Lower respiratory tract and can spread systemically.	Internalization by macrophages via phagocytosis and neutralizes lysosomes to prevent detection or lysis; able to survive indefinitely but erupts to cause infection when host is immunocompromised.^[^ ^150^, ^151^ ^]^
**Fungal**			
Aspergillus (mold; most common species *A. fumigatus)*	Fever, chills, shortness of breath, wheezing, headache, cough, (Rhinitis, bleeding of the lungs, systemic infection, and multi‐organ failure).	Upper and lower respiratory tract can spread systemically.	Can invade tissues by extending hyphae through endothelial and epithelial barriers.^[^ ^142^, ^168^ ^]^

### 3.1 Viral Pathogens

Viral infection may be replicated in vitro by incubating cell culture systems with an immunostimulant which mimics viral inflammatory processes such as polyinosinic:polycytidylic acid (Poly I:C),^[^ ^129^ ^]^ or by isolating live viruses and administering them in serum or aerosol deposition. Viral isolation first requires sampling and collection from an infected biological specimen, such as a nasal swab, which is then grown in vitro by infecting cells (typically mammalian cells), as viral replication requires a host. Media from infected cells can then be collected and separated from cells via filtration or centrifugation, as a source or virus particles.^[^ ^130^ ^]^ Common viral pathogens cultured in this way include Corona viruses, influenza, respiratory syncytial virus (RSV), and rhinoviruses which are listed in Table 4 and briefly discussed below.

**Figure 3:**
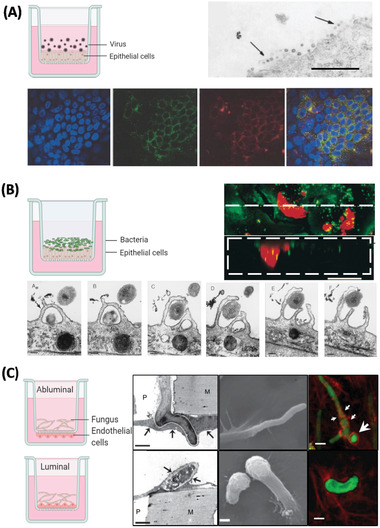
Examples of in vitro models used to study entrance and virulence mechanisms of A) viral, B) bacterial, and C) fungal pathogens. A) Apical entry and release of severe acute respiratory syndrome‐associated coronavirus in polarized Calu‐3 lung epithelial cells. Above: Transmission electron microscopy of release of SARS‐CoV virons from the apical surface of polarized Calu‐3 cells. Below: Colocalization of ACE‐2 and viral antigen in infected Calu‐3 cells, both ACE‐2 (green) and viral antigen (red) could be detected in infected cells. Importantly, both ACE‐2 and viral antigen appeared to colocalize in infected cells (yellowish). Reproduced with permission.^[^ ^92^ ^]^ Copyright 2005, ASM. B) Infection of primary human bronchial epithelial cells by *Hemophilus influenzae*. Above: Images collected by dual‐wavelength CLSM of cells infected for 3 h; colocalization of airway nuclei, bacteria (green) and vacuoles (red) can be seen in yellow, suggesting bacteria have been taken into the cells. Scale bar is 50 µm. Below: The series (A through F) demonstrates lamellipodia surrounding bacteria (black arrow) at the surface of a submerged airway cell culture after 4 h of infection. Reproduced with permission.^[^ ^141^ ^]^ Copyright 1999, ASM. C) Polarized response of endothelial cells to invasion by *Aspergillus fumigatus*. *A. fumigatus* hyphae invade the abluminal and luminal surface of endothelial cells by different mechanisms. Above: Hyphae invading the abluminal surface of endothelial cells, Arrows indicate an endothelial cell that is being invaded by a hypha. Below: Hyphae invading the luminal surface, arrows indicate endothelial cell pseudopods. Hyphae are shown in green and microfilaments in red. Bars represent 5 µm. Reproduced with permission,^[^ ^142^ ^]^ Copyright 2009, Wiley. The inset cartoon schematics represent the type of model chosen. Cartoon insets created using BioRender.com.

Corona viruses are classified into four types (alpha, beta, gamma, and delta) with Middle Eastern respiratory syndrome coronavirus (MERS‐CoV), severe acute respiratory syndrome Coronavirus (SARS‐CoV), and SARS‐CoV‐2; all of different lineages within the beta category. Corona viruses infect epithelial cells of the upper and lower respiratory tract via viral spike protein binding and cleavage by host cell proteases. MERS‐CoV enters via the CD26 receptor^[^ ^131^ ^]^ while both SARS‐CoV^[^ ^132^ ^]^ and SARS‐CoV‐2^[^ ^133^ ^]^ enter via the angiotensin converting enzyme 2 (ACE 2) receptor. In the case of novel pathogens, such as recently emerged corona viruses, it is essential to recapitulate infection in a representative in vitro model, to gain insight into the mechanisms of transmission, pathogenesis, and possible targets for vaccines. The specific mode of entrance and virulence have been studied in coronaviruses using cell lines,^[^ ^92^, ^134^ ^]^ primary cells,^[^ ^72^, ^134^ ^]^ and patient biopsies.^[^ ^135^ ^]^ For example, the apical entrance of coronavirus in bronchiole epithelial cells, via the ACE 2 receptor, has been shown by protein co‐localization in confocal Z‐Stack immunofluorescence imaging.^[^ ^92^ ^]^ Apical entrance and release of virions may also be demonstrated by sampling supernatant from apical and basolateral serum as well as via transmission electron microscopy imaging (**Figure** **3**A).

Influenza viruses infect epithelial cells of the upper respiratory tract, via binding of viral hemagglutinin to sialic acid containing receptors of target cells.^[^ ^136^ ^]^ The symptoms elicited following infection are due largely to the release of proinflammatory cytokine and chemokines for example interferons and tumor necrosis factor from viral‐infected cells. In vitro, emerging strains of influenza may be studied in order to elucidate replication and infectivity mechanisms as well as strain specific cytokine/chemokine profiles.^[^ ^87^, ^88^ ^]^

RSV infects cells of the lower respiratory tract via binding of viral fusion glycoprotein with Nucleolin containing surface receptors of target cells.^[^ ^137^ ^]^ RSV is easily transmitted and is a major cause of respiratory infection in children and infants. In vitro, the link between RSV virulence, airway hyperresponsiveness, and the production of specific cytokine profiles may be modeled using cell lines,^[^ ^138^ ^]^ organoids,^[^ ^85^ ^]^ or primary culture derived from pediatric patients populations.^[^ ^139^ ^]^ RSV infection of lung specific immune cells has also be used to study cross‐talk between immune cell and epithelial components for both pathogen virulence and protection mechanisms.^[^ ^70^ ^]^

Rhinoviruses are one of the most common causes of the common cold and exacerbations in lung disease such as asthma. Rhinoviruses have three species (A, B, and C) with infection occurring in epithelial cells of the upper respiratory tract. Rhinovirus induced asthmatic exacerbations may be modeled in vitro models by comparing healthy and asthma derived primary cells^[^ ^125^, ^140^ ^]^ or lung slices^[^ ^89^ ^]^ from patients and observing disease or patient specific inflammatory cytokine profiles as well as cell specific immune cell migration. Findings from in vitro studies such as these may then be replicated and correlated to in vivo investigations to assess translatability to the human condition, a principle factor in improving drug development and therapeutics in the clinical setting.

### 3.2 Bacterial Pathogens

Bacterial infection may be replicated in vitro by incubating cell culture systems with an immunostimulant which mimics bacterial inflammatory processes such as Lipopolysaccharides (LPS) or endotoxins.^[^ ^143^ ^]^ In contrast to a live virus, bacteria do not require host cells for replication, rather, growth and isolation of specific strains may be acquired using selective agar or media. Common respiratory bacterial pathogens cultured in this way include *Streptococcus pneumoniae*, *Mycobacterium tuberculosis*, and *Haemophilus influenzae* (Table 4).

*S. pneumoniae* commonly forms part of the upper respiratory tract flora and its presence is asymptomatic in most healthy individuals. However, under favorable environments or in compromised individuals, *S. pneumoniae* colonizes extracellular respiratory space, migrate to the lower respiratory tract and is the major cause of bacterial pneumonia in vulnerable patients. Virulence is associated with the release of invasion proteins such as pneumolysin, which contribute to host cell entrance and death via pore formation, toxin‐induced apoptosis or induction of host cell epigenetic changes.^[^ ^144^ ^]^ *S. pneumoniae* infection is also shown to decrease mucocilary clearance mechanisms and induce epithelial autolysis in primary respiratory organoid and biopsy samples.^[^ ^145^ ^]^ Cell line models have also been useful as a high‐throughput means for identifying novel targets and developing alternative treatments for resistant strains.^[^ ^146^ ^]^

Similar to *S. pneumoniae, H. influenzae* may be present in the upper respiratory tract flora and is an opportunistic pathogen, causing infection in vulnerable or immunocompromised individuals by migrating to the lower respiratory tract and/or systemically. Virulence is caused by surface Lipooligosaccharides and lipoproteins, which when attached to the mucosal surface, exert disruptive effects on cilia function.^[^ ^147^ ^]^ *H. influenzae* also produced proteases which help to evade macrophage induced lysis via mechanisms similar to that of *M. tuberculosis*. Virulence mechanism such as these have been studied in vitro by infecting cell lines.^[^ ^148^, ^149^ ^]^ It has also been shown that infection occurs via the rearrangement of epithelial cytoskeletons and micropinocytosis, demonstrated by microvilli and lamellipodia extending and engaging with bacteria, and the presence of bacteria within vacuoles of epithelial cells, respectively (Figure 3B).^[^ ^141^ ^]^

*M. tuberculosis* infects the lower respiratory tract and is the causative agent of tuberculosis. Infection occurs via macrophagic phagocytosis and contaminant neutralization of lysosomes. *M. Tuberculosis* is able to lie dormant within these cells, erupt when the host is immunocompromised and even cause chronic infection. Virulence of *M. Tuberculosis* is associated with the production of toxins, such as tuberculosis necrotizing toxin,^[^ ^150^ ^]^ encapsulation in a lipid containing coating, and participation in lysis‐evading mechanisms.^[^ ^151^ ^]^ Replication mechanisms have been studied in human alveolar cell lines^[^ ^152^, ^153^, ^154^, ^155^ ^]^ and in co‐culture with immune cell and ECM components.^[^ ^156^, ^157^ ^]^

In addition to complications caused by primary bacterial infections, viral infection also increases the risk of developing a secondary bacterial infection, termed a bacterial superinfection.^[^ ^47^ ^]^ Mechanisms behind this include viral‐induced desensitization of macrophages^[^ ^158^, ^159^ ^]^ and an impaired neutrophil and monocyte response.^[^ ^47^ ^]^ Viral‐induced epithelial damage may also facilitate the passage and colonization of bacterial pathogens within the respiratory tract and lung parenchyma. Furthermore, it is important to consider the respiratory microbiome in influencing a patient's susceptibility and response to infection. Many reviews exist which describe the complex interaction between the respiratory microbiome, epithelium, and immune system,^[^ ^160^, ^161^, ^162^, ^163^ ^]^ but in brief, the microbiome is influenced by a range of early life experiences such as mode of delivery, environment, diet, and respiratory infection. Additionally, the presence of underlying disease, immunosuppression, or certain drug treatment may influence microbiota profile, potentially leading to an inflammatory environment. Under these conditions, commensal microbial species may become pathogenic, such as those mentioned above, for example, *S. pneumoniae, H. influenzae*. Conversely, commensal respiratory bacteria may also have a protective effect. For example, *H. influenza*, which is a common cause of respiratory infection in children, may offer specific protective roles against developing RSV.^[^ ^164^ ^]^ Additionally, patient‐specific microbiota profiles have been linked to having protective affects against influenza infection and virulence.^[^ ^165^ ^]^ Therefore, the interaction between commensal and pathogenic microbes, within the respiratory system, are an important consideration when assessing patient specific responses to infection and therapeutics.

### 3.3 Fungal Pathogens

Like bacteria, fungal species may live in symbiosis with a host and, although possible to inhale infectious fungal agents, most infections are of the opportunistic type, developing disease mainly in immunocompromised individuals.^[^ ^166^ ^]^ Fungi replication occurs via spore spreading and, like bacteria, can be grown and isolated in vitro using selective agar or media.

*A. fumigatus* is the most common respiratory fungal species and is associated with development of aspergillosis. Aspergillosis may take a variety of forms. Allergic aspergillosis occurs when patients experience an allergic reaction to fungal spores and is most common in patients with underlying inflammatory lung conditions asthma and cystic fibrosis. Acute invasive aspergillosis on the other hand, is the most severe form of the disease and occurs in immunocompromised patients when the infection spreads systemically to other organs. Virulence of *A. fumigatus* occurs through the production of toxins such as Aflatoxin and Gliotoxin which exert immunosuppressive effects including disrupting cilia function, inhibiting phagocytosis, and inducing apoptosis.^[^ ^167^ ^]^ In vitro models of *A. fumigatus* have demonstrated Hyphae extensions are capable of penetrating pulmonary endothelial and epithelial cells as a mechanism of invasion.^[^ ^142^, ^168^ ^]^ Additionally, hyphae invasion induces a polarized response in endothelial cells, such that luminal invasion occurs via endocytosis and the formation of pseudopods, whereas abluminal invasion occurs via the disruption of microfilaments (Figure 3C).

The evidence provided thus far encompasses studies which use traditional in vitro models of respiratory infection; however, in the hope of providing more relevant, biomimetic, and high throughput drug discovery platforms, a range of more advanced and technology integrated model systems are continuously being developed. These are discussed in detail below.

## 4 Advances in Technology Integrated Models for Studying Host Pathogen Interaction

In parallel to the growing ethical concerns surrounding animal use in research and their lack of their clinical translatability,^[^ ^8^, ^9^, ^10^ ^]^ there has been a surge in the development of technology integrated 3D in vitro models which better reflect the human in vivo lung condition. For example, it is possible to integrate previously static 2D, 2.5D, and 3D models, for example, ALI co‐culture, organoids, etc. (as discussed in Section 4) with technological advances such as perfusion chambers^[^ ^73^, ^170^ ^]^ and lung‐on‐chips.^[^ ^101^, ^171^, ^172^ ^]^ Technology integrated biological systems have advanced knowledge surrounding the effect of culturing conditions and model architecture on relevant parameters such as cellular differentiation, immune cell recruitment, and cytokine profile, such that lung models are becoming increasingly, and more accurately, representative of the human condition. Here, we discuss the progression from perfusion bioreactor chambers to microfluidics and sensor integrated lung‐on‐chips, and how these have advanced our understanding of lung cell culture.

### 4.1 Lung‐on‐Chip

With the development of fluidics and commercially available perfusion chambers, it is possible to accelerate the speed of growth, differentiation, and development of 2D lung epithelial ALI models. Indeed, with perfusion systems, ciliogenesis, mucus production, and barrier formation are observed up to 14 days earlier when compared to static culture.^[^ ^73^ ^]^ Systems such as these enable the fast‐track addition of immune co‐culture and pathogen infection studies, significantly shortening experimental protocol times without sacrificing the complexity of a 3D ALI model. In parallel, the revolutionary development of microfluidic organ‐on‐chip technology during the last decade permits the coupling of microfluidics with microsensor technologies. Indeed, in addition to applying effective shear stress and flow, which enhances cellular differentiation,^[^ ^173^ ^]^ sample preparation, and delivery of nutrients,^[^ ^101^ ^]^ it is possible to integrate on‐chip biosensors such as pH sensors, microscopes, and electrodes.^[^ ^172^, ^174^ ^]^ In comparison to traditional culture systems, this enhances the speed of detection, breadth of readout data, device portability, and accelerates the point of care diagnostics.

**Figure 4:**
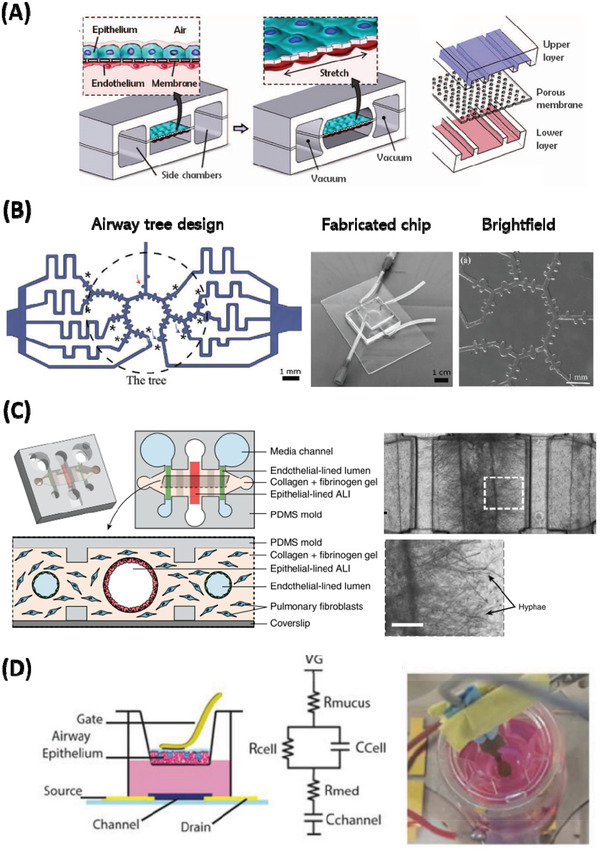
Examples of advanced technology integrated in vitro models of the lung showing A) mechanical actuation, B) complex microfluidic airway design, C) compartmentalization of lung components and infectious agents and D) advanced electronic monitoring of ALI culture. A) Compartmentalized PDMS microchannels form the alveolar‐capillary barrier. The device recreates physiological breathing movements by applying vacuum to the side chambers and causing mechanical stretching of the PDMS membrane. Reproduced with permission.^[^ ^171^ ^]^ Copyright 2010, AAAS. B) Anatomically inspired microfluidic acini‐on‐chip featuring an asymmetrical bipurification model of distal airways (blue arrows) and air‐ducts (red arrows). Reproduced with permission.^[^ ^177^ ^]^ Copyright 2019, Wiley. C) A microbial culture insert is inoculated with *A. fumigatus* on the left and *P. aeruginosa* on the right, facilitating volatile factor contact between the microbial cultures and air‐exposed center lumens lined with bronchiolar epithelial cells. Scale bar is 250 µm. Reproduced with permission.^[^ ^168^ ^]^ Copyright 2017, Nature Publishing Group. D) Effect of E Cigarette Emissions on Tracheal cells monitored at ALI using an organic electrochemical transistor. Integration of an ALI airway epithelium model into a flexible gate‐OECT platform for ALI resistance sensing, which conforms to the cell secreted mucus. Reproduced with permission.^[^ ^178^ ^]^ Copyright 2019, Wiley.

Organ‐on‐chips are commonly microfabricated from transparent and biocompatible polymers such as poly(dimethylsiloxane (PDMS) and poly(methlymethacrylate) (PMMA), via soft lithography‐based techniques, and consist of multiple layers of cell culture chambers integrated with microfluidic perfusion systems. A pivotal study carried out by Huh and colleagues in 2010 involved the development of a novel actuation system which mimicked breathing in the human lung.^[^ ^171^ ^]^ This “breathing lung‐on‐chip” consisted of an upper and lower chamber, separated by a thin porous PDMS membrane. The upper compartment contained human alveolar epithelial cells cultured at air interface, while the lower compartment contained human microvascular endothelial cells co‐cultured with human neutrophils under dynamic flow. Together, these compartments represented the alveolar capillary lung unit which, under the application of a vacuum in adjacent chambers, underwent cyclically stretching representative of physiological breathing (**Figure** **4**A). Importantly, this study demonstrated the effect of breathing on enhancing inflammatory and immune signaling in primary co‐culture. For example, when exposed to air pollutant extracts, an increase in pro‐inflammatory adhesion molecules and reactive oxygen species was observed, when compared to static cell culture. Similar lung‐on‐chip models, which utilize an actuated micro diaphragm to induce mechanical breathing, also demonstrate a breathing‐induced increase in barrier permeability, metabolic activity, and wound healing in lung epithelial cells.^[^ ^175^ ^]^ These examples highlight the importance of representing in vivo like breathing forces when assessing the extent of inflammation and immune cell activation that an airborne particle, pathogen, or drug candidate could have. Additionally, improvements in design and fabrication methods permit passive, rather than active, perfusion of chips. This reduces the need for additional external equipment and tubing, while improving reproducibility.^[^ ^176^ ^]^ Modern chip technology is also becoming increasingly compatible with standard characterization methods such as TEER, enzyme linked absorbency assays (ELISA), and permeability assays,^[^ ^176^ ^]^ making their integration into mainstream laboratories more amenable.

In another study, focusing on the effect of smoking induced respiratory inflammation and disease progression, a novel multi‐compartment robotic smoking machine was microengineered.^[^ ^103^ ^]^ This replicated all major aspects of physiological breathing including mechanical inhalation of smoke, controlled respiration parameters (respiration cycle, puff time, and inter puff interval), and flow rate over an air interfaced lung‐on‐chip. Compared to traditional exposure protocols, which deposit cigarette smoke extracts on top of cell culture, this study applied whole cigarette smoke under physiologically flow. This novel protocol revealed novel disease specific molecular signatures, potentially useful for future biomarker and drug target studies. Additionally, this study gave a detailed insight into smoke‐induced changes in ciliary beat frequency distribution, which may be linked to reduced mucociliary clearance observed in smokers. With advances in fabrication methods, it is also possible to create complex 3D microchannel networks, within lab‐on‐chip systems. Indeed, Schnirman and colleagues^[^ ^177^ ^]^ fabricated an anatomically inspired microfluidic model, mimicking the bifurcation networks of human alveolar tree structures which matched functional residue capacity values of pediatric populations (Figure 4B). Although complex structural models such as these are technically challenging to implement, they are fundamental in replicating and simultaneously studying the full breadth of cell types present in all parts of the airway. Models such those described above more fully recapitulate the human condition compared to traditional cell models and, although difficult to implement, are essential in reducing animal research and drug attrition rates.

Biomimetic 3D lung‐on‐chip models have also been increasingly applied to the study of pathogen invasion, disease‐associated inflammation, host–pathogen interaction, and therapeutic treatment of novel infectious agents. Unlike traditional in vitro models of pathogen invasion, which involve the direct measurement of pathogen‐induced effects upon epithelial layers, chip systems are able to physically compartmentalize and connect different cell and microbial populations. Thus, it is possible to study more complex interactions between the host and pathogen under physiological flow. It is also possible study the communication that occurs between the lung ECM, immune system, and circulating volatile compounds at ALI. Indeed, Barkal and colleagues^[^ ^168^ ^]^ microengineered an innovative bronchiole‐on‐chip device which contained a central airway lumen and adjacent endothelial lumens connected via a fibroblast‐collagen matrix (Figure 4C). A separate ”clickable” module, seeded with compartmentalized infectious microbials, was attached to the main lung unit. This was used to study pathogen‐derived volatiles on the respiratory epithelium. In this instance, co‐infection with the fungal and bacterial agents *A. fumigatus* and *P. aeruginosa*, respectively, was shown with hyphae extensions and leukocyte migration clearly observed at the site of infection (Figure 4C(III)). Lung‐on‐chip devices have also been used to study lung epithelial permeability^[^ ^172^, ^175^ ^]^ and single‐strain pathogen infection.^[^ ^163^, ^171^ ^]^ Pathogen‐induced effects on lung epithelial permeability may be measured via TEER or passage of fluorescently labelled molecules through the epithelium.^[^ ^172^, ^179^ ^]^ Additionally, cell effluent can be collected and assayed via ELISA or PCR for relative change in cytokine profile.^[^ ^163^ ^]^ In the case of co‐culture, immune cell migration to the site of infection may be observed via high resolution and real‐time microscopy imaging.^[^ ^171^ ^]^

As well as modelling inflammation and immune recruitment in healthy lungs, lung‐on‐chip devices are also used to model pathogen induced exacerbations in lung disease.^[^ ^63^, ^64^ ^]^ Diseased phenotypes may be modeled by directly culturing primary cells from diseased patients or alternatively, inflammation can be induced by stimulating cells with inflammatory proteins or cytokines implicated in disease pathology. For example, allergenic asthma‐like lung inflammation can be induced with the cytokine IL‐13, which is known to induce airway‐hyperresponsiveness and goblet cell hyperplasia in vivo.^[^ ^73^, ^158^ ^]^ These models can also be used as drug discovery platforms by applying novel therapeutics to microchannels and measuring effects on epithelial cell composition, function, and cytokine profile. Studies such as these, illustrate the importance of modelling complex aspects of the in vivo lung environment, such as physiological flow rate and breathing‐induced mechanical strain. Organ‐on‐chip technology paves the way for portable, multi‐parametric, and simultaneous assay platforms, which increasingly makes the study of respiratory pathogens in healthy and diseased human airways more efficient and accessible.

## 5 Future Directions

Although substantial progress has been made in recent years toward 3D and technology integrated in vitro lung models, there remain some limitations or problems to address. For example, in the case of organoid or complex scaffold structures, there is limited capability in monitoring the cellular components found within the core of the 3D systems. Secondly, in the case of ALI cultures, the present gold standards for monitoring epithelial integrity, such as TEER, require the apical surface to be submerged in an electrolyte/media. This negates the advantages of ALI culturing method, as well as preventing any long term/real‐time TEER measurements of any ALI culture. Some novel innovations and future prospective, which address these limitations, are highlighted below.

### 5.1 Conducting Polymer Scaffolds

As mentioned above, the use of polymer scaffolds and hydrogels in 3D cell culture has proven advantages such as increased viability, differentiation, and the ability to study cell–ECM interactions. However, there also remain limitations in accurately assessing/monitoring the inner portions of these 3D structures. One solution to this is the fabrication of complex cell architectures within conducting polymers, permitting the electronic monitoring of enclosed cell populations. Interestingly, in a 3D tissue engineered tubular model, the fabrication of conducting polymer scaffolds demonstrated the ability to monitor cell adhesion, growth and migration in real‐time, via material‐integrated electronic sensing abilities.^[^ ^180^ ^]^ This highlights the potential of scaffold systems to accurately monitor complex 3D architectures in a dynamic and mid‐throughput manner. One can see how this technology may be adapted or integrated into lung scaffolds for monitoring epithelial/endothelial permeability and immune cell adhesion/migration when performing pathogen challenge experiments. It is also possible to utilize hydrogels as biosensors by tuning them to detect pH, temperature, light, or electricity,^[^ ^181^ ^]^ which can be particularly beneficial for use in microfluidic devices for creating on‐chip readout systems. The field of bioelectronics, discussed below, looks promising for future application in monitoring cell and tissue culture in a non‐invasive, label free, and real‐time manner.

### 5.2 Advances in Flexible Electronics

A technology capable of conforming and electronically monitoring a range of complex 3D architectures, lies in the field of bioelectronics. Indeed, parallel to the rise of biocompatible and wearable electronic sensors in medical and commercial settings,^[^ ^182^, ^183^ ^]^ flexible electronics have also been implemented in a variety of in silico^[^ ^184^ ^]^, in vivo, and in vitro research applications.^[^ ^185^ ^]^ Of note are poly (3,4‐ethlyenedioxythiophene) doped (p‐type) with poly (styrene sulfonate) (PEDOT:PSS)‐based electrodes or organic electrochemical transistors (OECTs). The detailed physical theory of OECT operation is explored elsewhere,^[^ ^186^, ^187^ ^]^ but such technology has been integrated into a variety of biological formats, including Transwell ALI culture,^[^ ^178^ ^]^ planar and microfluidic devices,^[^ ^188^, ^189^, ^190^, ^191^ ^]^ PEDOT:PSS bio‐scaffolds,^[^ ^192^, ^193^ ^]^ self‐rolling sensors,^[^ ^194^ ^]^ and neuromorphic devices.^[^ ^195^ ^]^ In vivo examples include bioresorbable patches^[^ ^196^, ^197^ ^]^ and implantable electrocorticography devices for monitoring neuronal epileptiform discharge.^[^ ^198^, ^199^ ^]^ In each of these applications, OECT devices have shown superior performance when compared to conventional electrode recordings, including lower operational voltages, increased signal‐to‐noise ratio (SNR), and increased biocompatibility. Furthermore, OECTs display high capacitance, low impedance, mechanical flexibility, chemical tunability, and optical transparency, making them ideal candidates for multiparametric sensing, simultaneous characterization with optical techniques and improved efficiency, and accuracy of data acquisition.^[^ ^187^, ^200^, ^201^ ^]^ OECT devices have been used to study epithelial barrier formation and disruption,^[^ ^202^ ^]^ stem cell differentiation,^[^ ^203^ ^]^ and to detect analytes in human fluid samples for diagnostic purposes.^[^ ^204^ ^]^ In the line of pathogen infection, the application of OECTs have been largely used to study food‐borne or bacterial infection of intestinal^[^ ^205^ ^]^ and kidney cell lines.^[^ ^206^ ^]^ In relation to the respiratory epithelium, OECTs have been applied to the study of E‐cigarette aerosol exposure on human tracheal barrier integrity in ALI cultures^[^ ^178^ ^]^ (Figure 4D) and conductivity of ion channels implicated in pulmonary disease.^[^ ^207^ ^]^ Additionally, if biofunctionalized, OECTs, can achieve a high detection sensitivity of protein biomarkers,^[^ ^208^ ^]^ cell surface glycans,^[^ ^209^ ^]^ and human viruses^[^ ^210^ ^]^ which demonstrates the capability of this technology in advancing host–pathogen interaction studies.

## 6 Conclusions

Respiratory infection and related co‐morbidities are one of the leading causes of death worldwide, while also contributing a substantial socio‐economic burden. Given the recent SARS‐CoV‐2 pandemic, it has become increasingly evident that more efficient and biomimetic in vitro systems are needed to improve the efficacy, reproducibility, and translatability of therapeutics, antivirals, and vaccines. Here, we have given an overview of the biological and immunological components responsible for respiratory epithelial barrier integrity in health and disease. Furthermore, we have given an overview of the most common respiratory pathogens, as well as traditional 2D and more complex 3D in vitro models for studying host–respiratory pathogen interactions. Great improvements have been made in recent years in the fields of tissue engineering, material science, and biotechnology that have enabled the production of complex 3D models. For example, improvements in hydrogel composites have allowed for improved differentiation, proliferation, and longevity of cell/tissue culture. Developments in microfluidic and microfabrication techniques have also contributed crucial knowledge on the importance of mechanical, biochemical, and spatiotemporal cues in replicating an entire organ system. Additionally chip technology permits the integration of multiple biosensors in a compact design which offers advantages such as speed of processing, detection, breadth of readout data, and device portability. Finally, with the rise in the field of flexible electronic biosensors, which have the ability to physically conform to a range of complex 3D architecture, give multimodal, real time, and long term readouts, the future may see further integration of this technology with respiratory in vitro models.

## Conflict of Interest

The authors declare no conflict of interest.

## Author Contribution

S.L.B., S.J., and R.M.O, are responsible for the design of article contents. S.L.B, is responsible for the majority of the writing, design, and production of figures and tables. R.M.O. and J.S. provided feedback and guidance for the construction of the article. All authors have read the article and given approval.
